# Supplementary material for: AffyMAPSDetector: a software tool to characterize Affymetrix GeneChip™ expression arrays with respect to SNPs
Source: BMC Bioinformatics. 2007 Jul 30;8:276. doi: 10.1186/1471-2105-8-276 (PMC1959249; doi:10.1186/1471-2105-8-276)
Supplement: Additional file 13 — AffyMAPSDetector v1 source code. AffyMAPSDetector source code. [file 1471-2105-8-276-S13.zip › AffyMAPSDetector_v1_src/doc/affychip/snpdetector/model/Probe.html]

Probe


|  |  |  |  |
| --- | --- | --- | --- |
| **Overview** \| **Package** \| **Class** \| **Use** \| **Tree** \| **Deprecated** \| **Index** \| **Help** | | |  |
| **PREV CLASS**   **NEXT CLASS** | **FRAMES**    **NO FRAMES**     **All Classes** |  |  |
| SUMMARY: NESTED \| FIELD \| CONSTR \| METHOD | DETAIL: FIELD \| CONSTR \| METHOD |  |  |


---


## affychip.snpdetector.model Class Probe

```
java.lang.Object
  affychip.snpdetector.model.Probe
```

**All Implemented Interfaces:**: java.lang.Comparable

---

public class **Probe** extends java.lang.Object implements java.lang.Comparable

Title: Probe SNP Detector

Description: Affymetrix Gene-Probe SNP Detection

A gene-probe on an Affy expression chip. A probe occupies one feature (i.e.
one spot or one cell) on the microarray chip and it is probe is uniquely
identified by a combination of:  

1. Probe-set ID
2. X Coordinate of the cell
3. Y Coordinate of the cell

This class represents a 25mer oligo probe in a gene sequence whose 13th
position corresponds to mis-match postion.

Copyright: Copyright (c) 2004

Company: George Mason University

**Version:**
:   1.1

**Author:**
:   Sunita Kumari

---

**Constructor Summary**

`Probe(java.lang.String probesetId,
java.lang.String seq,
java.lang.String x,
java.lang.String y,
int interrogation)`


|  |  |
| --- | --- |
| **Method Summary** | |
| `int` | `compareTo(java.lang.Object o)` |
| `int` | `getInterrogationPosition()` |
| `java.lang.String` | `getKey()` |
| `int` | `getMax()` |
| `int` | `getMiddlePosition()` |
| `int` | `getMin()` |
| `java.lang.String` | `getProbsetId()` |
| `java.lang.String` | `getRange()` |
| `java.lang.String` | `getSeq()` |
| `java.lang.String` | `getSnpList()` |
| `java.lang.String` | `getX()` |
| `java.lang.String` | `getY()` |
| `boolean` | `hasSNPs()` |
| `void` | `setMax(int max)` |
| `void` | `setMiddlePosition(int middle)` |
| `void` | `setMin(int min)` |
| `void` | `setSNPs(java.lang.String[] snpList)` |
| `java.lang.String` | `toString()` |

**Methods inherited from class java.lang.Object**

`clone, equals, finalize, getClass, hashCode, notify, notifyAll, wait, wait, wait`

**Constructor Detail**

### Probe

```
public Probe(java.lang.String probesetId,
             java.lang.String seq,
             java.lang.String x,
             java.lang.String y,
             int interrogation)
```


**Method Detail**

### setSNPs

```
public void setSNPs(java.lang.String[] snpList)
```

---


### hasSNPs

```
public boolean hasSNPs()
```

---


### getSnpList

```
public java.lang.String getSnpList()
```

---


### getInterrogationPosition

```
public int getInterrogationPosition()
```

---


### getMiddlePosition

```
public int getMiddlePosition()
```

---


### setMiddlePosition

```
public void setMiddlePosition(int middle)
```

---


### getMin

```
public int getMin()
```

---


### setMin

```
public void setMin(int min)
```

---


### getMax

```
public int getMax()
```

---


### setMax

```
public void setMax(int max)
```

---


### getProbsetId

```
public java.lang.String getProbsetId()
```

---


### getX

```
public java.lang.String getX()
```

---


### getY

```
public java.lang.String getY()
```

---


### getSeq

```
public java.lang.String getSeq()
```

---


### getKey

```
public java.lang.String getKey()
```

---


### getRange

```
public java.lang.String getRange()
```

---


### toString

```
public java.lang.String toString()
```

---


### compareTo

```
public int compareTo(java.lang.Object o)
```

:   **Specified by:**: `compareTo` in interface `java.lang.Comparable`


---


|  |  |  |  |
| --- | --- | --- | --- |
| **Overview** \| **Package** \| **Class** \| **Use** \| **Tree** \| **Deprecated** \| **Index** \| **Help** | | |  |
| **PREV CLASS**   **NEXT CLASS** | **FRAMES**    **NO FRAMES**     **All Classes** |  |  |
| SUMMARY: NESTED \| FIELD \| CONSTR \| METHOD | DETAIL: FIELD \| CONSTR \| METHOD |  |  |


---
